# Outcomes of Congenital Nasolacrimal Duct Obstruction Surgery Converted into Balloon Dilation and Silicone Intubation due to Probing Difficulty

**DOI:** 10.1155/2022/4045789

**Published:** 2022-03-12

**Authors:** Oren Yaakov Sagiv, Achia Nemet, Asaf Achiron, Doron Neumann, Raimo Tuuminen, Oriel Spierer

**Affiliations:** ^1^Sackler Faculty of Medicine, Tel Aviv University, Tel Aviv, Israel; ^2^Pediatric Ophthalmology and Adult Strabismus Unit, Edith Wolfson Medical Center, Holon, Israel; ^3^Helsinki Retina Research Group, University of Helsinki, Helsinki, Finland; ^4^Department of Ophthalmology, Kymenlaakso Central Hospital, Kotka, Finland

## Abstract

**Background:**

To report the outcomes of balloon catheter dilatation and silicone intubation as a sequential secondary surgery under the same anesthesia, a stepwise approach for congenital nasolacrimal duct obstruction (NLDO) when probing and irrigation as primary procedure fails.

**Methods:**

A retrospective study included children with NLDO who underwent probing and irrigation only, and those who underwent in the same surgery under anesthesia, adjunct balloon catheter dilation and silicone intubation due to difficulty of the probe passage or fluid regurgitation from the punctum. The primary outcome was surgical success defined as resolution of preoperative symptoms and signs at 1 month.

**Results:**

A total of 105 NLDO cases were included. Eighty-four cases underwent probing and irrigation only, whereas 21 cases required balloon dilation and silicone intubation consecutively after the first procedure. Patient age at surgery was higher for those requiring balloon dilatation and intubation (30.3 ± 8.0 months) when compared to those with probing and irrigation only (22.4 ± 10.3 months, *p* < 0.001). The onset of symptoms, preoperative clinical findings regarding tearing and discharge and gender distribution of patients were comparable between the two groups. During the follow-up, the overall success rate for probing and irrigation only was 76.2% (64 out of 84 cases) and for balloon dilatation and silicone tube intubation was 90.5% (19 out of 21 cases).

**Conclusions:**

The surgical team may prepare to proceed with secondary surgery under the same anesthesia after the initial attempt of probing and irrigation. This stepwise two-stage approach in patients with congenital NLDO failing primary surgery resulted in a high success rate with minimal interventions, avoiding repeated general anesthesia.

## 1. Introduction

Congenital nasolacrimal duct obstruction (NLDO) is a common condition of infancy and early childhood, in which there is a failure in the development of the nasolacrimal duct drainage system. Clinically, the patient will have an overflow of tears, discharge, and mild palpebral inflammation. The prevalence of NLDO ranges from 5% to 20% in the early phase of childhood, but more than 90% of cases will resolve before the age of 1 year [1].

The pathogenesis of NLDO lies in a mechanical obstruction located distally in the nasolacrimal duct at the valve of Hasner, where this structure enters the nose. The main causes of obstruction are a pathological persistence of the membrane at the distal portion of the nasolacrimal duct, bone abnormalities, or a stenosis of the inferior meatus leading to a narrowing in the lacrimal drainage system [2]. Several studies have shown that in most cases, the obstruction tends to naturally and spontaneously resolve within the first year of life [3, 4]. Nevertheless, in some cases, this disorder may persist beyond the first year of life, and in this case, a surgical intervention is needed.

The most common first-line treatment is probing and irrigation of the nasolacrimal system with a 75–90% success rate [5]. However, another surgery, such as balloon catheter dilatation and/or silicone intubation may be required. The preference of the procedures may depend on the patient's age at the time of surgery [6]. As the child is younger there is a tendency towards probing and irrigation only, and as the child is older, a balloon catheter dilation and/or silicone intubation may be primarily added [2]. Nevertheless, other surgeons will use balloon or silicone tube only when a second procedure is needed after primary probing and irrigation have failed.

Here, we started the primary surgery with probing and irrigation of the nasolacrimal system. If both probing and irrigation go smoothly, the operation is terminated. Otherwise, the surgeon continues in the same surgery to perform balloon nasolacrimal duct dilation and silicone intubation. In the current study, we aim to report the baseline characteristics and clinical outcome of this protocol for NLDO and to assess whether using a stepwise two-stage approach is effective.

## 2. Methods

### 2.1. Study Participants and Data Collection

A retrospective chart review was performed on all patients that underwent surgery for congenital NLDO between March 2008 and December 2018, at the E. Wolfson Medical Center, Sackler Faculty of Medicine, Tel Aviv University. Only patients who underwent their first surgery for NLDO were included. The study was approved by the Internal Review Board of the E. Wolfson Medical Center and complied with the principles outlined in the Declaration of Helsinki.

### 2.2. Surgical Technique

Prior to the surgery, the parents were discussed about the stepwise two-stage approach and gave their consent for balloon catheter dilation followed by silicone intubation if needed. Under general anesthesia, a lacrimal dilator was used to dilate the inferior punctum and a lacrimal probe was then gently threaded through the lacrimal sac and into the bony canal and nasal cavity. The procedure was repeated through the superior punctum. Methylene blue-labeled saline [7] was then flowed into the nasal cavity, which was confirmed with suction. If the obstruction was easily relieved by the probing procedure and methylene blue-labeled saline was flowing freely into the nasal cavity, the surgery was terminated.

When the passage of the lacrimal probe was more difficult than usual or there was regurgitation from the punctum, then, balloon catheter dilation followed by silicone intubation was performed. The 2 mm balloon (LacriCATH®, QUEST Medical, Allen, TX, USA) was inserted through the inferior punctum to the distal nasolacrimal duct and inflated to 8 atm for 60 seconds, then deflated, pulled a bit out and reinflated for 30 seconds. The balloon was then deflated and removed from the nasolacrimal system. Canalicular intubation (Monoka de Fayet-Bernard, FCI Ophthalmics, Pembroke, MA, USA) was then performed—typically one tube was placed through the inferior punctum and a second tube through the superior punctum. In some cases, only one tube was placed in the inferior or in the superior punctum. The tubes were recovered from the nose and trimmed. At the end of the procedure combined neomycin/polymyxin B and dexamethasone ointment (Maxitrol®, Novartis, Basel, Switzerland) was applied to the surface of the operated eye. A combination of neomycin/polymyxin B and dexamethasone drops (Maxitrol®, Novartis) was prescribed in the operated eye 4 times a day for 1 week. The tubes were left in place for 3 months and then removed in the clinic [8]. All surgeries were performed by one surgeon (D.N.).

### 2.3. Main Outcome Measure

Treatment outcome was determined one month after the probing and irrigation or after removal of the tubes. Surgical success was determined as the resolution of preoperative symptoms and signs (tears, discharge, and eyelid inflammation).

### 2.4. Statistical Analysis

The data were analyzed using the Minitab software (version 17, Minitab Inc., Paris, France). Normal distribution was assessed by the Shapiro–Wilk test. For continuous variables with a normal distribution an independent *t*-test was used and the Mann–Whitney *U* test was used for non-normally distributed variables. For categorical variables, Fisher's test was used. A ranked stepwise regression analysis was performed to analyze the impact of different variables on operation success. The association among various characteristics with operation success was examined with logistic regression analysis. *p* values less than 0.05 on a two-sided test were considered statistically significant.

## 3. Results

### 3.1. Demographic and Preoperative Data

A total of 105 NLDO cases (73 patients, 50.5% female) were included in the study. The mean age at surgery was 23.9 ± 10.4 months (range, 2–52 months). Ninety-three (88.6%) cases had tearing and discharge as a sign of NLDO at the baseline examination and 12 (11.4%) cases had tearing only. Eighty-four (80.0%) cases underwent probing and irrigation, and 21 (20.0%) cases underwent in addition balloon catheter dilation followed by silicone intubation. No intraoperative or postoperative complications were documented. Patients that underwent probing and irrigation only were younger than patients who required balloon catheterization and silicone intubation (22.4 ± 10.3 months vs. 30.3 ± 8.0 months, *p*=0.001, Table 1). Baseline characteristics of the patients are presented in Table 1.

### 3.2. Surgical Outcomes

The overall success rate was 79.0% (83 out of 105 cases). The success rate in the probing and irrigation group and in the balloon and silicone group was 76.2% and 90.5%, respectively (*p*=0.35, Table 2). In 17 out of 21 cases (80.9%) in which a silicone tube was placed, the tube was present at the 3-month follow-up visit. Table 2 depicts pre- and intraoperative parameters as surgical success predictors among all 105 cases included in this study. None of the parameters included such as age at the onset of symptoms, gender, and clinical presentation had a significant impact on the surgical success rate.

When comparing preoperative and intraoperative factors and success rate between both groups, none of the abovementioned parameters (age at the onset of symptoms, gender, and clinical presentation) had an impact on the surgical outcomes (Table 3). A multivariable logistic regression model including gender, age at the onset of symptoms, age at surgery, and type of surgery could not identify any predictive variables associated with surgical success (*p*=0.45, data not shown). No correlation was found between the evaluated parameters and operation success: probing and irrigation vs. balloon and silicone (*p*=0.765, exp(*B*) = 0.478), procedure location: inferior punctum vs. superior punctum vs. both (*p*=0.918, exp(*B*) = 0.914), surgery complications: simple vs. complex (*p*=0.060, exp(*B*) = 5.16), using a balloon at the surgery (*p*=0.708, exp(*B*) = 0.405). Collectively, complete data analysis reveals a 10.3% percentage of variance (*p*=0.279).

## 4. Discussion

In this study, the success rate was 76.2% in children who underwent probing and irrigation only, and 90.5% in children who underwent adjunct balloon catheter dilatation and silicone tube intubation at the same anesthesia due to probing and irrigation failure. Although the study groups were biased having the primary surgery more complicated in children with balloon catheter dilatation and silicone tube intubation, the success rates were high in both groups. Instead of having two separate general anesthesia in complicated surgeries, our study emphasizes the stepwise two-stage approach under the same anesthesia as preferable surgical practice. This approach resulted in high success rates, with no operative complications or adverse events during the follow-up. Furthermore, demographic and clinical parameters did not affect surgical success rates.

Congenital NLDO typically becomes symptomatic in the first month of the child's life [1], as it was also found in our study. Infants with NLDO present with tearing and debris on their eyelashes, mild redness of the lower eyelid, increase in the size of the tear meniscus, epiphora, and recurrent eye infections [1]. Most of the congenital NLDO cases resolve spontaneously by the first year of life. For children for whom symptoms do not resolve by then, surgical intervention should be considered. Lacrimal duct probing is the first-line surgical procedure [9, 10]; however, no consensus has been reached as to whether this should always be the preferred treatment, regardless of the child's age and probing difficulty. Probing, which is done by inserting a small blunt probe into the punctum and throughout the lacrimal drainage system, has several advantages over more complex procedures such as short surgical time, minimal surgical manipulation, low risk of bleeding, and no need for tube removal later on. In addition, evidence supports the efficacy and safety of both in-office based surgery and operating room surgery for congenital NLDO [11]. Yet, treating bilateral NLDO in the operating room may be better [11]. In our practice, we always perform these procedures in the operating room. The main disadvantage of simple probing is a failure rate of up to 25% of all cases [5]. Similar to other studies, our failure rate was 23.8%.

A surgical alternative is to proceed during the primary surgery with nasolacrimal duct intubation [12, 13]. Silicone tubes may prevent adhesions, constrictions, and restenosis after the probing. The tubes are usually removed in the office after one to six months [14]. Reported success rates of this procedure vary between 85% and 96% when used as primary treatment [14, 15]. Our success rate of 90.5% is in line with previous reports. The main advantages of tubing are high success and low complication rates. The disadvantages of this procedure are additional surgical manipulations with a longer anesthesia time and the need for an additional procedure of tube retrieval after the surgery. Blunt retrieval of the tube from the nose during the surgery may occasionally be challenging and can take time. A common postoperative complication is premature loss of the silicone tube, which was reported to occur in 3% to 44% of cases [14]. Our 19.1% incidence of premature tube loss is comparable to previous studies. It was reported that success rates were not significantly different between planned tube removal and premature tube extrusion [8].

Balloon catheter dilation is yet another alternative for primary surgery. This procedure involves probing the nasolacrimal duct with a semiflexible wire probe containing an inflatable balloon. Originally, this procedure has been recommended as the initial treatment for congenital NLDO in children older than 24 months [15]. The reported success rate of this procedure ranges from 77% to 91% [16, 17]. The main advantage of the balloon catheter is its action as a nasolacrimal duct dilator without the need for a second procedure later in the office. It is also a shorter procedure and technically easier than silicone intubation. Its main disadvantage is the high cost of the disposable balloon catheter used for the treatment [18].

Arora et al. reported a success rate of 78% in children aged <36 months that underwent probing procedures [19]. The success rate declined to 50% in children aged over 36 months. In such cases, nasolacrimal intubation or balloon catheter dilation were preferred as primary surgery. In contrast, Robb et al. did not find differences in probing success rates between children aged younger or older than 36 months [20]. Gunton et al. found similar success rates between probing and balloon catheter dilatation as the primary treatment for congenital NLDO [21]. Both procedures had a high success rate (86% for probing and 90% for balloon catheter dilatation), that were not diminished in children older than 36 months. Similarly, Goldich et al. did not find a significant difference in the surgical outcomes of probing as compared to balloon catheter dilatation, except for patients who had a relatively narrow nasolacrimal bone duct [22]. In general, we agree with the approach that age is not a determinant factor in the decision whether to perform balloon dilatation and silicone intubation in the primary surgery for NLDO. Therefore, the surgical approach should be based on the intraoperative findings, specifically probing difficulty, and the presence of regurgitation from the punctum during irrigation, which resemble the complexity of the nasolacrimal anatomy. In our study, there was a variance among the patients' age, with some children older than 36 months. As our clinic is also a referral center, some patients were referred quite late by their ophthalmologist or their pediatrician. Some pediatricians advise the parents to wait for a spontaneous resolution, and the parents wait until the child has grown up.

The retrospective nature of this study is its main limitation. The large difference in the sample size between the two groups is another limitation. In addition, nasal endoscopy, which may help identify the cause of difficulty in probing or failure of the procedure, was not performed. However, our 90.5% success rate in the difficult cases where balloon dilation and silicone tube intubation were performed is comparable to the reported success rates in cases where endoscopic-assisted probing was performed [23]. Furthermore, nasal endoscopy is more expensive, extends anesthesia time, has a learning curve, and in some cases necessitates the help of an otolaryngologist. As the patients who underwent balloon catheter dilatation and silicone tubing were older, we cannot rule out age bias which might influence the surgeon's interpretation of the findings during surgery. It is also possible that long-lasting signs in older children cause chronic infection and fibrosis. As a result, the passage of the lacrimal probe is more difficult than usual.

In patients with complex congenital NLDO, late probing may have a lower success rate than intubation [24], although, in a large systematic review, success rates did not differ between immediate or deferrer probing, balloon dilatation, or intubation, and between mono- or bicanalicular intubation [25]. Nevertheless, the novelty in our surgical strategy lies in the fact that decision of which procedure to choose was not age-dependent but was based on surgical findings. This approach, i.e., the decision of whether to continue with stent intubation based on the intraoperative anatomical findings, was also described in adults undergoing endoscopic dacryocystorhinostomy [26]. This surgical strategy has importance in minimizing anesthesia time, especially in older children where probing and irrigation goes smoothly, and so, additional surgical steps are avoided. It also has the advantage of avoiding additional surgery and anesthesia in younger children where probing and irrigation do not go smoothly during the first surgery, and the surgeon continues with balloon dilation and intubation. A prospective study randomizing patients who appear to be functionally or anatomically obstructed following probing and irrigation into two arms, those in which the surgery is terminated and those who receive sequential balloon catheter dilatation and silicone tubing at the same anesthesia, will give a more definite answer to the question posed. Another potential prospective approach is to randomize patients in whom NLDO was easily relieved by the probing procedure into two arms, those in whom the surgery is terminated at that point and those who receive balloon catheter dilatation and silicone intubation as an adjunct therapy at the same anesthesia. These studies are of importance in terms of costs and risks involved in a case when probing and irrigation alone are deemed to have residual obstruction, based on the possible additional success rate when balloon catheter dilatation and silicone tube intubation are added to the first procedure.

In conclusion, both approaches, probing and irrigation alone and balloon catheter dilation and silicone tube intubation, are adequate treatments for persistent NLDO. Here, our results emphasize that based on intraoperative probing and irrigation difficulty, the surgeon may use his clinical judgment to decide to proceed in the same anesthesia with balloon catheter dilation and silicone tube intubation after the initial attempt of probing and irrigation. This surgical process, including preoperative planning, instrumentation, and discussion with the parents prior to the operation, should be anticipated in case the probing reveals a narrow nasolacrimal duct or irrigation shows regurgitation from the punctum. In that case, the surgical team should be able to consider performing a balloon dilation and silicone intubation under the same general anesthesia, regardless of the child's age. This stepwise method is clinically preferred and most probably a cost-effective approach for the treatment of congenital NLDO.

### 4.1. Key Messages

The most common first-line nasolacrimal duct obstruction surgery is probing and irrigation with a 75–90% success rate.

Our results emphasize that based on intraoperative probing and irrigation difficulty, and regardless of the child's age, conversion of the probing and irrigation in the same anesthesia into balloon catheter dilation and silicone tube intubation resulted in a high success rate with minimal interventions and good compliance.

This stepwise two-stage approach is most probably a cost-effective approach for the treatment of congenital nasolacrimal duct obstruction.

## Figures and Tables

**Table 1:** Baseline characteristics between patients with probing and irrigation and those with balloon catheter dilation and silicone tubing.

		Probing and irrigation (*n* = 84)	Balloon and silicone tube (*n* = 21)	*p* value
Age at surgery (months)		22.4 ± 10.3	30.3 ± 8.0	0.001
Gender (female)		40 (47.6%)	13 (61.9%)	0.33
Onset of symptoms at the first month of life		73 (86.9%)	19 (90.5%)	1.00

Symptoms	Tearing only	5 (6.0%)	5 (23.8%)	0.12
	Tearing & discharge	79 (94.0%)	16 (76.2%)	

Probing	Superior punctum	9 (10.7%)	4 (19.0%)	0.34
	Inferior punctum	50 (59.5%)	9 (42.9%)	
	Superior & inferior punctum	25 (29.7%)	8 (38.1%)	

Data are given as mean ± SD or absolute numbers (and proportions).

**Table 2:** Success rates among patients who underwent surgery for nasolacrimal duct obstruction.

		Success rate (%)	*p* value
Onset of symptoms	At the first month of life	81.1	0.71
	After the first month of life	76.9	

Gender	Female	77.4	1.00
	Male	80.8	

Symptoms	Tearing only	90.0	0.68
	Tearing & discharge	80.2	

Silicone tube position	Superior punctum	76.9	0.86
	Inferior punctum	81.0	
	Superior & inferior punctum	83.9	

**Table 3:** Effect of patient-related factors on surgery success rates (%) between patients with probing and irrigation and those with balloon catheter dilation and silicone tubing.

		Probing and irrigation (*n* = 84)	Balloon and silicone tube (*n* = 21)	*p* value
Overall success rate (%)		76.2	90.5	0.35

Onset of symptoms	At the first month of life	78.9	89.5	0.29
	After the first month of life	72.7	100	0.40

Gender	Female	78.9	84.6	0.66
	Male	77.3	100	0.13

Symptoms	Tearing only	100	80.0	0.29
	Tearing & discharge	77.3	98.3	0.15

## Data Availability

The data used to support the findings of the study are available upon request.
